# Dietary Inflammatory Index Is Related to Heart Failure Risk and Cardiac Function: A Case–Control Study in Heart Failure Patients

**DOI:** 10.3389/fnut.2021.605396

**Published:** 2021-04-06

**Authors:** Jalal Moludi, Nitin Shivappa, Soghra Alisgharzadeh, James R. Hébert, Mohammad Alizadeh

**Affiliations:** ^1^School of Nutrition Sciences and Food Technology, Kermanshah University of Medical Sciences, Kermanshah, Iran; ^2^Clinical Research Development Center, Imam Reza Hospital, Kermanshah University of Medical Sciences, Kermanshah, Iran; ^3^Cancer Prevention and Control Program, University of South Carolina, Columbia, SC, United States; ^4^Department of Epidemiology and Biostatistics, Arnold School of Public Health, University of South Carolina, Columbia, SC, United States; ^5^Connecting Health Innovations LLC, Columbia, SC, United States; ^6^Student Research Committee, Tabriz University of Medical Sciences, Tabriz, Iran; ^7^Nutrition Research Center, Faculty of Nutrition, Tabriz University of Medical Sciences, Tabriz, Iran

**Keywords:** dietary inflammatory index, dietary intake, inflammation, case–control studies, heart failure

## Abstract

**Aims:** Previous studies suggest that diet and inflammation are important risk factors for heart failure (HF); however, the associations remain unclear. The Dietary Inflammatory Index (DII^®^) was established to measure the inflammatory capacity of individuals' diet. This study aimed to explore the DII in HF subjects compared with controls.

**Methods and Results:** We conducted a case–control (116 cases and 113 controls) study that recruited in the similar clinics. DII scores were calculated based on dietary intakes. N-Terminal pro-brain natriuretic peptide (NT-proBNP) levels and ejection fraction (EF) were assessed in both groups. In order to analyze DII scores with HF as the outcome, we used conditional logistic regression. A linear regression was applied to explore the associations between the DII and left ventricular EF (LVEF).

There was statistically significant difference in DII scores in cases vs. controls (−0.16 ± 1.37 vs. −0.33 ± 1.67; *p* = 0.040). Conditional logistic regression has shown that subjects with higher DII scores had higher risk of HF. For every one-point rise in DII score, the odds of having HF increased by 30% (OR: 1.30; CI: 1.03, 1.69; *p* = 0.047). The EF was inversely associated with saturated fatty acid (β = −0.34, 95% CI: −0.61, −0.07; *p* = 0.012). Subjects with higher DII scores had higher NT-proBNP levels and had lower EF.

**Conclusion:** The DII score was associated with high probability of HF. It appears that consumption of anti-inflammatory diet may lead to the prevention of HF and therefore suggests that dietary modification with the goal of reducing DII scores could be a valuable strategy for improving clinical outcomes in these patients.

## Background

Heart failure (HF) affects an estimated 23 million people worldwide (1) and leads to substantial numbers of hospitalizations and health-care costs. Therefore, prevention of HF has become a major public health concern, not just a major cause of its increasing prevalence but also because of its deleterious effect on quality of life (2). Low-grade inflammation is a principal factor leading to development of HF (3, 4). Furthermore, erythrocyte sedimentation rate (ESR), as a marker of inflammation, was a significant predictor of HF (3, 5). In particular, it has been shown that some environmental factors, such as dietary intake, are important in triggering an inflammatory response (6, 7).

The relationship between diet and HF is well-established. Healthy dietary patterns such as the Mediterranean diet are independently connected with a lower possibility of all-cause cardiovascular and cancer-related mortality (8, 9). Several studies have shown that certain nutrients can help alleviate diseases and modify systemic inflammation (9–12). On the other hand, diets with high sugar, refined starches, and saturated and trans-fatty acids and are poor in antioxidants and fibers may cause an activation of chronic inflammation (13). Undeniably, dietary patterns have long been known as potent factors leading to the acceleration of HF pathogenesis (14). Many dietary components have long been supposed to play a vital role in development of inflammation via both anti-inflammatory and pro-inflammatory mechanisms. Therefore, it is important to develop a scoring algorithm that takes into account these influences within an overall dietary pattern. Although most of the studies focus on whole diet in underlying mechanisms leading to HF development (15), knowledge about dietary components of that specific dietary components is still limited especially because the pathways leading to HF are not fully understood.

The dietary inflammatory index (DII^®^) has been designed to quantify the potential inflammatory properties of a diet (16). The association between DII and various inflammatory markers has been established by multiple cohorts (17, 18). A large growing body of data investigating the associations between DII and risk of a wide range of non-communicable diseases including obesity, diabetes, cancers, and cardiovascular disease (CVD) has emerged (18–21).

The DII is not just restricted to macronutrients and micronutrients; it also includes flavonoids and frequently consumed parts of the diet such as tea and spices. The potential interactions among nutrients also must be taken into account (22). Moreover, individuals with greater DII had more risk of metabolic disorders (19, 23). As start and development of HF is related to a chronic pro-inflammatory state (24), we hypothesized that higher DII scores (or higher DII quartiles) are associated with HF and worse clinical outcomes. Based on previous research with the DII and evidence linking inflammation with CVD, this study aimed to examine the relationship between the DII and HF in a case–control research conducted in Iran.

## Methods

### Subjects

The current hospital-based matched case–control study was conducted from January 2017 through February 2018 in cardiac medical centers in Tabriz, East Azerbaijan Province, Iran. Subjects with HF were recruited by convenience sampling from the Madani Heart Center under the supervision of Tabriz University of Medical Sciences. The hospital is a single specialty heart hospital, which is the largest of its kind in the northwest of the country, with 330 administrative and clinical staff.

The sample size calculation has been explained before. Briefly, the sample size was designed by the following formula: *n* = [(Zα/2 + Zβ)2 × {(p1 (1–p1) + (p2 (1–p2))}]/(p1–p2)^2^, where p1 is the proportion of the subjects with low DII, α-error = 0.05, and power = 80% (1–β) (25). Consequently, a sample size of 120 patients was calculated for the study (120 in each group). We also assumed 5% loss (125 + 5), and the final sample size of 250 was included in this study.

Patients with HF clinically defined based on the European Society of Cardiology guidelines, who were established less than a year before enrolment, were involved in the study. Other inclusion criteria were subjects aged 30–70 years and did not announce modifications to their diets from the time during diagnosis. We included only new cases in the study with the intention of lessening the possibility that subjects altered their diet considerably in during diagnosis. Patients were diagnosed to have HF if they had had left ventricular ejection fraction (LVEF) ≤ 0.35, were in New York Heart Association (NYHA) functional classification class II to IV HF for ≥ 3 months before study, had a heart rate ≥ 68 bpm, and were receiving standard therapy of diuretics and an angiotensin-converting enzyme (ACE) inhibitor or digoxin. HF cases were patients who had two-dimensional echocardiography and confirmed by clinical symptoms. Also, the patients with preserved EF but with clinical symptoms were also considered in this study. Exclusion criteria in the case group included the following: (a) non-adherence to the study protocol, (b) reporting caloric intake >4,000 or <800 kcal/day, (c) inability to respond to the questions, and (d) supplement therapy for HF.

**Figure 1:**
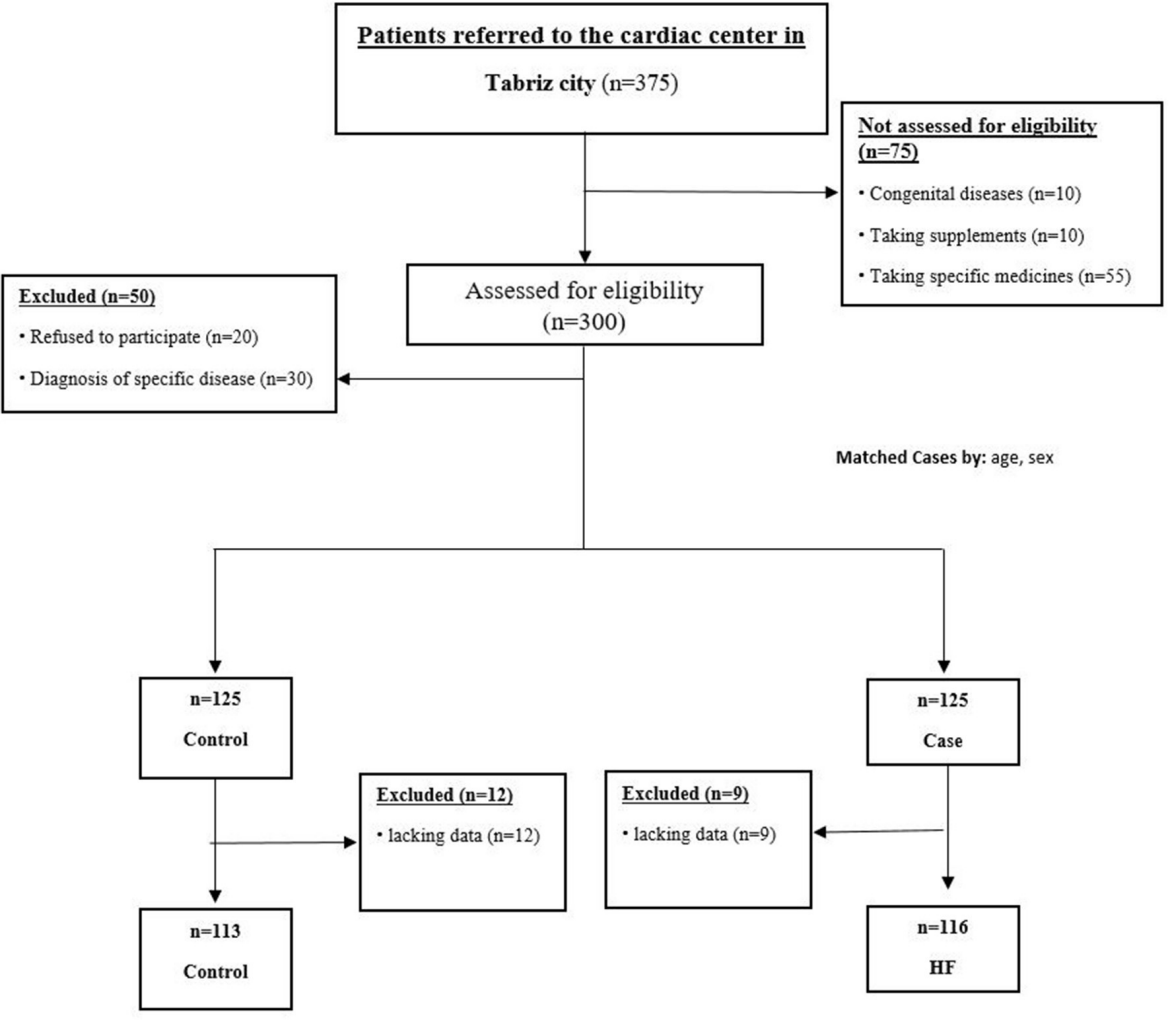
Flowchart of study.

Controls (*n* = 125) were randomly selected from the same hospital who were admitted to the hospitals and underwent percutaneous coronary intervention (PCI) and no evidence of cardiomyopathy (EF ≥ 40) but who might have hyperlipidemia and hypertension (*n* = 125). Exclusion criteria for controls were prior diagnosis of inflammatory diseases of the peripheral nervous system; cancer; liver diseases and gastrointestinal, metabolic, and endocrine disorders; and immune system disorders and have a special diet (e.g., vegetarian or weight-loss diet) and low cardiac function (EF <40). Patients with acute myocardial infarction (MI) and decreased cardiac function were excluded. Controls were coordinated to cases on age (±4 years) and sex. All of the patients were screened by an expert cardiologist for eligibility. Of joined cases and controls, nine HF and 12 controls were excluded because of lacking food frequency questionnaires (FFQs) or other data. The final sample comprised 116 cases and 113 controls, demonstrating a 90% response rate (Figure 1). Informed consent was obtained from all subjects. The research was approved by the research council of the Tabriz University of Medical Sciences (No. IR.TBZMED.REC.1397.184). Information on sociodemographic characteristics, anthropometric data, biochemical variables, and medical history was assessed using a standard questionnaire by a trained interviewer.

### Assessment of Anthropometric Data

We used a scale (Seca 770; Seca GmbH & Co, Hamburg, Germany) with 0.5-kg accuracy while patients were wearing light clothing and no shoes in order to measure the subjects' body weight. We also utilized a tape with 0.5-cm accuracy in order to assess their height. We computed the body mass index (BMI) by weight-to-height ratio (BMI = kg/m^2^).

N-Terminal pro-brain natriuretic peptide (NT-proBNP) levels was assessed by the quantitative electrochemiluminescence immunoassay (ELISA) method (Roche kite).

### Determination of Ejection Fraction

EF was measured by two-dimensional echocardiography. The participants were divided into EFs ≥40% (controls) and <35% (HF case) subgroups. Furthermore, we used clinical symptoms, which confirmed classification.

### Dietary Assessment

We obtained dietary intake by making use of a 138-item semi-quantitative FFQ involving of a list of a finite foods and beverages with standard serving sizes usually consumed by Iranians (26, 27). Patients were requested to state how often they consumed each of the food listed by the number of times (every day, weekly, once a month, and annually). According to a standard serving size for each nutrient item, and then each contributor's intake was transformed to weight equivalents (i.e., μg, g, and mg) per day. Then, dietary information was investigated for energy and nutrients, using revised Nutritionist IV software (Nutritional Database Manager 4.0.1, First Data Bank, USA). The development and validation of the DII are described in detail elsewhere (22). The greater the DII score, the more pro-inflammatory the diet. More negative values represent the more anti-inflammatory diets. The DII tertile cut points were categorized based on cut points: Tertile 1 ≤ −1.132; Tertile 2 = −1.132 to 0.467; Tertile 3 ≥ 0.467.

### Data Analysis

The data were examined by SPSS 16 software (SPSS Inc., Chicago, IL, USA). Data are presented as mean and standard deviation, frequency and percent, or median and interquartile range. The normality of data was assessed and proved by skewness and kurtosis test. For all statistical assessments, *p* < 0.05 was considered as significant. Independent samples *t*-test and Mann–Whitney *U*-test were used for between-group differences. *p*-values for trends were calculated for variables across DII tertiles. Conditional logistic regression was done to calculate odds ratio (OR) and 95% confidence intervals (CIs) for HF as the outcome. Multiple linear regressions via the enter method were used to evaluate the associations between EF as outcome and continuous outcomes such as DII, sex, age, and BMI.

## Results

Table 1 indicates the distribution of HF and controls with reference to some variables. DII scores in our study ranged from −3.3 (maximum anti-inflammatory score) to +2.82 (most pro-inflammatory score). By design, the demographic distributions were not different in cases and controls. Furthermore, the control group had higher values of high-density lipoprotein (HDL) (*p* = 0.001) and lower level of low-density lipoprotein (LDL) than the case groups (*p* = 0.01).

**Table 1:** Characteristics of patients in the case and control groups (*n* = 229).

**Characteristics**	**Cases (*n* = 116)**	**Controls (*n* = 113)**	***p*-value**
Age, years	56.34 ± 12.99	57.40 ± 13.43	0.547
**Gender, n (%)**			
Male	75 (64.66)	69 (61.06)	0.573
Female	41 (35.34)	44 (38.94)	
BMI, kg/m^2^, mean ± SD	26.63 ± 5.75	26.73 ± 5.29	0.892
**Smoking, n (%)**			
Yes	35 (30.17)	22 (19.47)	0.061
No	81 (69.83)	91 (80.53)	
**Hypertension, n (%)**			
Yes	61 (52.59)	51 (45.13)	0.259
No	55 (47.41)	62 (54.87)	
EF (%)	25.34 ± 9.70	43.3 ± 19.5	0.001
LDL, mg/dl, mean ± SD	131.60 ± 30.14	120.37 ± 34.84	0.010
HDL mg/dl, mean ± SD	39.47 ± 7.26	44.81 ± 9.15	0.000
Cholesterol mg/dl, mean ± SD	180.89 ± 47.24	173.11 ± 46.43	0.210
TG, mg/dl, mean ± SD	151.34 ± 48.62	139.90 ± 48.26	0.075
DII, mean ± SD	−0.16 ± 1.37	−0.33 ± 1.67	0.040

*Student t-test was used for continuous variables. Chi-square test was used for categorical variables. Significance was at p < 0.05. p-values are based on Mann–Whitney U-test*.

*DII, dietary inflammatory index; BMI, body mass index; EF, ejection fraction; LDL, low-density lipoprotein; HDL, high-density lipoprotein; TG, triglycerides*.

The results also indicated that the mean DII score for the HF group was higher than that of the control group (−0.16 ± 1.37 vs. −0.33 ± 1.67; *p* = 0.040). When DII scores were transformed toward tertiles (Table 2), cumulative trends across tertiles of DII were detected for total fat and saturated fat, whereas statistically substantial decreasing trends were perceived for some anti-inflammatory intake such as fiber and beta carotene. However, in the analysis via the DII shown as tertiles, we failed to find any significant trend of increasing risk (*p* > 0.05) for other cardiovascular risk factors including lipid profile. Also, when DII was transformed into tertiles according to DII, there are some differences observed between the micronutrient intake of the participants (Table 3).

**Table 2:** Characteristics of the participants according to the quartiles of dietary inflammatory index.

**Variables**	**Tertile 1**	**Tertile 2**	**Tertile 3**	***p*_**trend**_**
**Demographic**				
Age (years)	56.79 ± 10.44	56.89 ± 14.90	56.98 ± 14.13	0.995
Gender, n (%)				0.735
Male	46 (61.33)	48 (63.16)	48 (64.00)	
BMI (kg/m)	26.43 ± 4.71	27.09 ± 6.38	26.61 ± 5.42	0.752
Smoking, n (%)	24 (32.00)	17 (22.37)	15 (20.00)	0.090
**Pro-inflammatory**				
Total fat (g)	71.05 ± 30.13	84.75 ± 41.19	94.23 ± 44.92	<0.001
Cholesterol (mg)	231.44 ± 155.77	202.39 ± 98.96	194.97 ± 108.23	0.198
Saturated fat (g)	19.00 (9.00, 23.19)	17.39 (9.74, 20.53)	30.70 (18.53, 33.41)	<0.001
**Anti-inflammatory**				
Fiber (g)	18.41 ± 8.82	4.00 ± 1.44	13.35 ± 5.73	<0.001
Beta carotene (μg)	267.17 (97.72, 451.10)	73.16 (23.49, 519.60)	146.00 (17.51, 296.66)	<0.001

*For abbreviations, see Table 1. Values are presented as mean ± SD for variables with normal distribution or median (interquartile) for variables without normal distribution*.

*Tertile 1 ≤ −1.132; Tertile 2 = −1.132 to 0.467; Tertile 3 ≥ 0.467*.

**Table 3:** Micronutrient intake of the participants according to the tertiles of dietary inflammatory index.

**Variables**	**Tertile 1**	**Tertile 2**	**Tertile 3**	***p*_**trend**_**
Sodium (mg)	1115.00 (775.10, 2038.00)	1119.50 (499.00, 1973.00)	1999.00 (1367.00, 2131.00	0.020
Iron (mg)	14.51 (1,232, 14.94)	13.11 (9.20, 16.95)	12.87 (11.80, 17.88)	0.147
Zinc (mg)	7.91 (5.81, 10.75)	7.37 (5.17, 10.54)	9.15 (7.31, 1,032)	0.513
Magnesium (mg)	254.7 (181.27, 272.43)	195.50 (151.60, 232.70)	202.87 (183.70, 213.50)	0.006
Manganese (mg)	2.18 (1.88, 2.46)	1.93 (1.39, 2.48)	1.60 (1.26, 2.11)	0.401
Potassium (mg)	2728.00 (2375.00, 3636.00)	2067.00 (1696.50, 2466.67)	2588.5 (2318.00, 2676.00)	0.044
Calcium (mg)	694.67 (509.33, 1054.00)	663.30 (389.67, 817.50)	679.00 (526.30, 1077.00)	0.969
Phosphorus (mg)	1012.00 (785.40, 1255.00)	989.65 (611.87, 1,113.00)	1109.00 (1055.00, 1148.00)	0.589
Copper	1.34 (1.09, 1.47)	0.99 (0.77, 1.46)	1.09 (0.87, 1.22)	0.005
Selenium	0.10 (0.09, 0.14)	0.11 (0.08, 0.15)	0.08 (0.05, 0.17)	0.048
Chromium	0.05 (0.03, 0.08)	0.04 (0.02, 0.06)	0.05 (0.02, 0.07)	0.209
Vitamin A	802.67 (686.10, 922.70)	416.00 (271.80, 843.80)	422.30 (352.5, 502.33)	<0.001
Vitamin E	4.79 (2.98, 7.24)	3.57 (2.09, 5.56)	3.57 (2.51, 6.08)	0.031
Vitamin K	70.28 (44.90, 114.40)	30.67 (18.29, 50.66)	40.48 (19.05, 74.33)	<0.001
Vitamin D	1.00 (0.21, 1.99)	1.05 (0.04, 2.00)	0.79 (0.03, 2.11)	0.634
Thiamin	2.28 (1.65, 2.46)	1.63 (1.31, 2.07)	1.60 (1.48, 1.92)	<0.001
Riboflavin	1.56 (1.11, 1.92)	1.42 (0.87, 1.56)	1.52 (1.48, 1.83)	0.268
Niacin	19.88 (17.21, 24.98)	16.78 (12.83, 21.00)	17.79 (14.34, 22.89)	0.060
Pantothenic acid	4.92 (4.09, 5.64)	3.83 (2.52, 4.81)	3.94 (3.83, 4.57)	<0.001
Pyridoxine	1.38 (1.08, 2.27)	0.98 (0.80, 1.44)	1.11 (0.93, 1.45)	<0.001
Folate	210.40 (186.13, 251.60)	135.00 (101.28, 245.20)	155.20 (131.67, 199.20)	<0.001
Cobalamin	2.83 (2.31, 3.34)	2.83 (1.66, 3.70)	3.51 (1.98, 4.08)	0.474
Biotin	23.94 (17.11, 26.48)	15.37 (10.98, 21.95)	16.84 (11.52, 21.48)	<0.001

*Data are median (interquartile range)*.

*Tertile 1 ≤ −1.132; Tertile 2 = −1.132 to 0.467; Tertile 3 ≥ 0.467*.

Clinical markers by DII tertiles are presented in Table 4. Especially, members in tertiles 3 had higher NT-proBNP and had lower EF (Table 4). The present results led us to conclude that low DII score leads to better clinical outcome in HF patients. However, in the analysis via the DII shown as tertiles, we failed to find any significant trend (*p* > 0.05) for other cardiovascular risk factors including lipid profile.

**Table 4:** Distribution of some important clinical markers across categories of DII.

**Variables**	**Tertile 1**	**Tertile 2**	**Tertile 3**	***p*_**trend**_**
EF	43.12 ± 24.94	37.08 ± 14.23	33.11 ± 12.76	**0.044**
LDL (mg/dl)	123.05 ± 31.68	128.03 ± 33.62	126.96 ± 34.36	0.627
HDL (mg/dl)	42.07 ± 9.22	43.00 ± 8.50	41.37 ± 8.35	0.515
Cholesterol (mg/dl)	174.64 ± 41.75	169.46 ± 46.93	187.80 ± 50.11	0.371
TG (mg/dl)	145.81 ± 52.24	149.43 ± 44.22	141.17 ± 49.83	0.582
NT-proBNP	109.81 ± 47.24	101.53 ± 43.12	155.17 ± 59.21	**0.036**

For abbreviations, see Table 1. Values are presented as mean ± SD for variables with normal distribution or median (interquartile) for variables without normal distribution.

*Tertile 1 ≤ −1.132; Tertile 2 = −1.132 to 0.467; Tertile 3 ≥ 0.467*.

*Bold values are statistically significant*.

Logistic regression analysis was done with HF as the outcome (Table 5), and the findings showed that each unit increase in DII score was associated with a 30% (OR: 1.30; CI: 1.03, 1.69; *p* = 0.047) increase in the odds of being diagnosed with HF. Significant OR also was perceived for triglyceride (TG), LDL, and HDL.

**Table 5:** Conditional logistic regression analysis with HF status as outcome.

**Variables**	**Odds ratio**	**95% CI**	***p*-value**
DII	1.303	1.003, 1.693	0.047
HDL	1.127	1.060, 1.198	<0.001
TG	0.988	0.979, 0.997	0.008
LDL	0.985	0.972, 0.998	0.034
Saturated fatty acids	0.905	0.861, 0.951	<0.001

*For abbreviations, see Table 1*.

*Adjusted for BMI and smoking*.

In a linear regression model including LVEF as the outcome, EF value was inversely associated with saturated fatty acid (β = −0.34, 95% CI: −0.61, −0.07; *p* = 0.012) (Table 6). No statistically significant relationship was established between the DII and the LVEF value. Finally, the set of variables including DII, HDL, TG, LDL, and saturated fatty acid significantly explained 9% of variance in EF status [*F*_(9, 215)_ = 3.54, *p* ≤ 0.001].

**Table 6:** Summary of linear regression analysis based on EF as outcome.

**Variables**	**B**	**SE**	**95% CI**	***p*-value**
DII	0.73	0.85	−1.12, −0.03	0.389
HDL	0.28	0.13	0.01, 0.55	0.038
TG	−0.08	0.02	−0.12, −0.03	0.001
LDL	−0.06	0.03	−0.13, 0.01	0.090
Saturated fatty asides	−0.34	0.13	−0.61, −0.07	0.012
Adjusted *R*^2^	0.09			

*For abbreviations, see Table 1. Adjusted for age, sex, BMI, and smoking*.

## Discussion

The current case–control study applied the DII score to the dietary intake of patients with HF for the first time. We detected that the DII score was related to increased risk of HF, thus implicating a pro-inflammatory diet in its etiology. However, EF was not associated with DII score.

It has been claimed that inflammation has a significant impact on the pathogenesis of CVDs and that higher high-sensitivity C-reactive protein (CRP) concentration accelerates the development of HF (28). Similarly, plasma levels of tumor necrosis factor-α (TNF-α) and IL-6 also predict HF outcomes (29). In addition, as beginning and development of HF accompany the chronic pro-inflammatory state, extra pro-inflammatory dietary patterns are associated with increased HF incidence and worse clinical outcome (30). One potential mechanism for the apparent relationship between the DII score and high risk of inflammatory diseases like CVD is the impact of diet on the cytokines levels, which regulate inflammatory response. Inflammatory cytokines also play a vital role in the underlying pathophysiological processes of HF (31).

While there are many studies supporting the concept of the protective effect of diets, including approaches to stop hypertension [Paleolithic, Dietary Approaches to Stop Hypertension (DASH) diet, low carb diet, vegetarian diet, and low-fat diet] on the prevention of HF, their effectiveness is not clear yet. DASH and Mediterranean diets revealed a protective impact on the HF incidence and/or deteriorating on EF (13, 32), but these results need to be able to replicate and investigate other diets (such as low DII) and to test the generalizability in post-MI patients. In other words, the consumption of vegetables and fruits has been proven to decrease inflammation, whereas the consumption of foods like meat and butter increases inflammation through increasing levels of CRP (33, 34). Indeed, focusing on the entire diet by computing DII score, which takes many categories of dietary components into account, more precisely indicates the relationship between diet and the risk of HF.

Previous findings have shown the relationship between the DII score and incidence of CVD (MI, stroke, and CVD death) (19). Numerous studies have indicated the significant role of chronic inflammation as an important factor that interferes in the development of CVD. In this context, dietary patterns are adjustable factors that have a huge potential to exert a powerful anti-inflammatory or pro-inflammatory effect (35). We observed a higher DII score in HF subjects compared with controls. This result is consistent with a previous systematic review of studies, which found that the DII scores were inversely associated with cardio-metabolic risk factors and CVDs (36). Therefore, a diet including pro-inflammatory components such as saturated and trans fat can cause proliferation of MI, oxidative stress (37, 38), and inflammation, which can cause ventricular remodeling and possibly HF development.

Our finding is consistent with previous result for DII and chronic disease in many clinical settings. Previously, we observed a negative association between DII and EF, which agrees with a previous SUN (“Seguimiento University of Navarra”) cohort study that was conducted in 18,794 individuals with 8.9 years' follow-up, where the number of new CVD events cases in the highest DII score was 2.03 times more than the lowest quartile (39). In another cohort study, carried out on 7,216 subject (55–80 years) at high risk of cardiovascular events, the highest quartile (most pro-inflammatory diet) was reversely associated with the number of CVD incidences (OR = 1.73; 95% CI: 1.15, 2.60) (40). A comparable negative relationship was reported between HF risk and DII score in patients with previously diagnosed CVDs (OR = 0.31; 95% CI: 0.12, 0.82; *p* = 0.018) (41). In contrast, the SU.VI.MAX study included 7,743 participants (aged 35–60 years) after 11.4 years' follow-up; no associations were observed for DII scores and the composite CVD outcomes (32). Effect sizes for HF risk have mostly been in the range of nearly OR 1.5–3, which is similar to our results. By comparing the results from this study, we hope to determine the association between DII and HF. The DII scores also in current population were comparable with those of earlier studies. The DII based on FFQ in our study population seems to be acceptable for this type of examination and for interpretation of the results in relation to earlier results. The DII score was highly positively associated with the intakes of saturated fatty acid. The higher intakes of saturated fatty acid are associated with inflammatory properties (5, 31); so it is not unexpected that there was a correlation with HF as reported by DII.

Numerous investigations have revealed a positive correlation between lipid profile (including total cholesterol, LDL, and HDL) and risk of HF and clinical outcomes (42, 43). The lack of a significant association in our study may be partially a result of the low levels of these outcomes among participants at baseline; also 60% of patients were under statin therapy. Low serum total cholesterol is related with increased mortality in patients with HF (43). In particular, HDL cholesterol was proposed to exert anti-inflammatory and antioxidant activities, which would lessen the pro-inflammatory state in patients with HF (44). Indeed, an association among HDL cholesterol, HF, and DII may be explained on the basis of the inflammation hypothesis (24), which suggests that low DII score modulates inflammatory immune function and also that HDL cholesterol has antioxidant and anti-inflammatory properties, both of which can further reduce risk of HF.

The potential cardiovascular effects of several foods and dietary patterns are still incompletely understood. Some research has suggested that “Western” dietary patterns, which contain high intakes of saturated and trans fatty acids, and low-fruit/low-vegetable intake, result in higher CRP levels and increased risk of CVD event (5, 10, 31, 40). In this regard, it seems that the dietary patterns with low DII scores prevent the development of HF.

Previously, the DII has been revealed to be related with different inflammatory markers including homocysteine and CRP (9). Diets having high amounts of vegetables and fruits are related with low levels of hs-CRP (34). Certain nutrients such as magnesium, fiber, and omega-3 fatty acids are associated with low levels of inflammation (14). A further novel finding is that those higher in DII had higher NT-proBNP and had lower EF, which mean low intensity of HF. The NT-proBNP levels were measured in all patients, and they were followed up and had a well-accepted biomarker of cardiac function (45). The present work led us to conclude that low DII score is associated with better cardiac function in HF patients.

This is the first study in the world to explore the relationship between the DII score and HF. Despite its strengths, our study had several limitations. First, we did not quantity inflammatory biomarkers, and as a result, we were incapable of evaluating this association. The second limitations relate to the generalizability of our results because of its design, which does not permit the distinction between cause and effect based on temporality. Therefore, we were unable to determine if a pro-inflammatory dietary pattern and higher DII score cause HF development or whether the existence of HF resulted in a more pro-inflammatory diet, which in turn resulted in higher DII. Thus, perspective studies, including intervention trials, are required to determine the role of dietary intakes as a cause of HF more firmly.

## Conclusion

In summary, the DII has presented a valuable means for evaluating the potential inflammatory of diets in HF patients. This study on HF patients conducted in Iranian subjects indicated a possible role of diet through the development of inflammation. Our results proposed evidence suggesting that an upper DII score (representative an extra pro-inflammatory diet) is strictly connected with the occurrence of HF. These results propose the significance of promoting dietary intake with low DII score for the subjects at risk for HF. Future studies, including intervention trials, are required to find this association precisely. The significance of this work is of major importance for public health-care planners, since it would test the low inflammatory diet hypothesis at population basis and may provide an additional, non-pharmacologic means for the prevention of HF.

## Data Availability Statement

The raw data supporting the conclusions of this article will be made available by the authors, without undue reservation.

## Ethics Statement

The studies involving human participants were reviewed and approved by the research council of the Tabriz University of Medical Sciences (No: IR.TBZMED.REC.1397.184). The patients/participants provided their written informed consent to participate in this study.

## Author Contributions

JM and MA: conceived and designed the experiments. JM performed the experiments. SA, JM, and NS: analyzed the data. All authors: wrote the paper.

## Conflict of Interest

JH owns controlling interest in Connecting Health Innovations LLC (CHI), a company that has licensed the right to his invention of the dietary inflammatory index (DII) from the University of South Carolina in order to develop computer and smart phone applications for patient counseling and dietary intervention in clinical settings. NS was an employee of CHI. The remaining authors declare that the research was conducted in the absence of any commercial or financial relationships that could be construed as a potential conflict of interest.

## Abbreviations

ANOVA: analysis of variance
CAD: coronary artery disease
CRP: C-reactive protein
CVD: cardiovascular disease
NT-proBNP: N-terminal (NT)-pro hormone BNP
LVEF: left ventricular ejection fraction
ESR: erythrocyte sedimentation rate
DII: Dietary Inflammatory Index
HF: heart failure
HDL: high-density lipoprotein cholesterol
LDL: low-density lipoprotein cholesterol
NYHA: New York Heart Association
TC: total cholesterol
TNF-α: tumor necrosis factor-α.
